# Transcriptome-wide identification and characterization of genes exhibit allele-specific imprinting in maize embryo and endosperm

**DOI:** 10.1186/s12870-023-04473-8

**Published:** 2023-10-06

**Authors:** Xiaomei Dong, Haishan Luo, Wenjing Bi, Hanyu Chen, Shuai Yu, Xiaoyu Zhang, Yuxin Dai, Xipeng Cheng, Yupeng Xing, Xiaoqin Fan, Yanbin Zhu, Yanling Guo, Dexuan Meng

**Affiliations:** 1 College of Bioscience and Biotechnology, Shenyang Agricultural University, Shenyang, 110866 Liaoning China; 2 Shenyang City Key Laboratory of Maize Genomic Selection Breeding, Shenyang, 110866 Liaoning China; 3 College of Agronomy, Shenyang Agricultural University, Shenyang, 110866 Liaoning China; 4 Manas Agricultural Experimental Station of Xinjiang Academy of Agricultural Sciences, Changji, 832200 Xinjiang China; 5 National Key Laboratory of Maize Biological Breeding, Key Laboratory of Genetics and Breeding of Main Crops in Northeast Region, Ministry of Agriculture and Rural Affairs, Liaoning Dongya Seed Industry Co., Ltd, Shenyang, Liaoning, 110164 China

**Keywords:** Maize, Allele-specific imprinting, Embryo, Endosperm, Kernel development

## Abstract

**Background:**

Genomic imprinting refers to a subset of genes that are expressed from only one parental allele during seed development in plants. Studies on genomic imprinting have revealed that intraspecific variations in genomic imprinting expression exist in naturally genetic varieties. However, there have been few studies on the functional analysis of allele-specific imprinted genes.

**Results:**

Here, we generated three reciprocal crosses among the B73, Mo17 and CAU5 inbred lines. Based on the transcriptome-wide analysis of allele-specific expression using RNA sequencing technology, 305 allele-specific imprinting genes (ASIGs) were identified in embryos, and 655 ASIGs were identified in endosperms from three maize F1 hybrids. Of these ASIGs, most did not show consistent maternal or paternal bias between the same tissue from different hybrids or different tissues from one hybrid cross. By gene ontology (GO) analysis, five and eight categories of GO exhibited significantly higher functional enrichments for ASIGs identified in embryo and endosperm, respectively. These functional categories indicated that ASIGs are involved in intercellular nutrient transport, signaling pathways, and transcriptional regulation of kernel development. Finally, the mutation and overexpression of one ASIG (*Zm305*) affected the length and width of the kernel.

**Conclusion:**

In this study, our data will be helpful in gaining further knowledge of genes exhibiting allele-specific imprinting patterns in seeds. The gain- and loss-of-function phenotypes of ASIGs associated with agronomically important seed traits provide compelling evidence for ASIGs as crucial targets to optimize seed traits in crop plants.

**Supplementary Information:**

The online version contains supplementary material available at 10.1186/s12870-023-04473-8.

## Background

Diploid sexually reproducing organisms inherit an allele of each gene from both parents, masking the deleterious effects of recessive mutations in heterozygotes. Nevertheless, a subset of genes in flowering plants and mammals are subject to imprinting, whereby genes are expressed preferentially from one parental allele. Genomic imprinting is restricted primarily to the embryo and endosperm in plants [1]. Triploid endosperm tissue (with two maternal and one paternal genome) develops along with the diploid embryo (with one maternal and one paternal genome) and is essential for normal embryo patterning and growth in maize.

Transcriptomic analysis of the kernel that develops from F1 hybrids is an effective approach to identify imprinted genes in flowering plants. Several dozen to hundreds of imprinted genes have been detected and characterized in various plants, including Arabidopsis, rice, maize, sorghum, caster bean, A. lyrata, Capsella rubella, rape seed, wheat, and sunflower [2–14]. The first imprinted gene, the *R* gene, exhibits allele-specific imprinting in maize [15]. Maternal bias in *R* expression causes mottled, reduced levels of kernel anthocyanins when *R* is inherited through pollen. However, the mottled phenotype associated with imprinting is absent in the non-imprinting R allele (*R-sc:124*) [16]. The *dzrl* posttranscriptionally controlling the accumulation of 10-kDa zein in the maize endosperm is also an allele-specific imprinted locus [17, 18]. Hence, some examples of imprinting are allele-specific, in that only certain alleles of these loci are imprinted. Some studies also found that several imprinted genes are imprinted variants by comparing imprinted genes identified in reciprocal crosses constructed from multiple inbred lines by transcriptomic analysis [19–22]. In Arabidopsis, while most genes had the same imprinting pattern in endosperm from six different crosses representing three sets of reciprocals, 12 genes were imprinted differently depending on whether they were inherited from the male or female of a given strain [20]. In rice, imprinted genes were compared among three pairs of reciprocal hybrids, including reciprocal hybrids between subspecies, japonica intra-subspecies, and indica intra-subspecies [22]. A total of 546 imprinted genes in inter-subspecies hybrids, 211 imprinted genes in japonica intra-subspecies hybrids, and 286 imprinted genes in indica intra-subspecies hybrids were identified. Only five imprinted genes were commonly detected in all pairs of reciprocal hybrids. In maize, approximately 10% of imprinted genes are variably imprinted in the endosperm tissue of five reciprocal hybrid pairs [19]. As noted above, imprinting can also be variable within species, meaning that a subset of genes is maternally expressed gene (MEG) or paternally expressed gene (PEG) when a certain strain is the male or female parent. Cis genetic or trans genetic or epigenetic variation is associated with imprinting variation [20].

The traits controlled by imprinted genes reflect the genotype of a single parent. To date, although functional studies of imprinting genes are limited, the important role of imprinting genes has been found in many biological processes of seed development [23, 24], postzygotic interploidy hybridization, and germination processes [25–30]. Mutations in some imprinted genes lead to a distinct phenotype of seed defects [31–34]. For example, *Fl3* (*Floury3*), a MEG in maize endosperm, encodes the plant AT-rich sequence and the zinc binding protein (PLATZ). *fl3* mutants showed severe defects in endosperm development and significantly reduced seed dry weight [35]. The *defective18* (*de18*) gene of endosperm, a PEG in rice, maize, and Arabidopsis, is required for auxin biosynthesis. Auxin plays a necessary role in normal endosperm proliferation in Arabidopsis [36], and *de18* positively regulates endosperm proliferation in maize [37]. Furthermore, known studies in rice, Capsella, and Monkey flower (*Mimulus*) have reported that unsuccessful imprinting has been associated with failed interspecific and interploidy hybridization [30, 38]. Imprinted genes also play a role in the regulation of germination processes and that preferential maternal allelic expression can implement maternal inheritance of seed dormancy levels [28]. Therefore, the study of the maize imprinted gene is of great significance both in theory and application.

Due to significant heterotic performance and the well-known complex genome, maize is an ideal model system for analyzing imprinting variants [39–41]. In this study, using RNA sequencing technology, a combination of statistical significance and proportion filters was implemented to identify and classify MEGs and PEGs in the embryo and endosperm of three hybrid F1 crosses. Comparison of imprinting in three reciprocal crosses within maize reveals allelic variation for imprinting. Further functional analysis indicated that these ASIGs may make important contributions to kernel development. Knockout mutant of one ASIG influence the length and width of the kernel. This study provides valuable resources for the konwledge of ASIGs in maize or even cereals.

## Results

### Identification of genes exhibiting allele-specific imprinting in maize embryo and endosperm

To identify genes exhibiting allele-specific imprinting (ASIG) in maize kernels, the inbred lines CAU5, B73 and Mo17 were selected to generate reciprocal crosses, B73 × Mo17 and Mo17 × B73 (denoted as BM/MB), B73 × CAU5 and CAU5 × B73 (denoted as BC/CB), and Mo17 × CAU5 and CAU5 × Mo17 (denoted as MC/CM). In our previous work, RNA sequencing (RNA-seq) of the immature embryo and endosperm at 11 days after pollination (DAP) from BC/CB, MC/CM and BM/MB was performed [42].

**Fig. 1:**
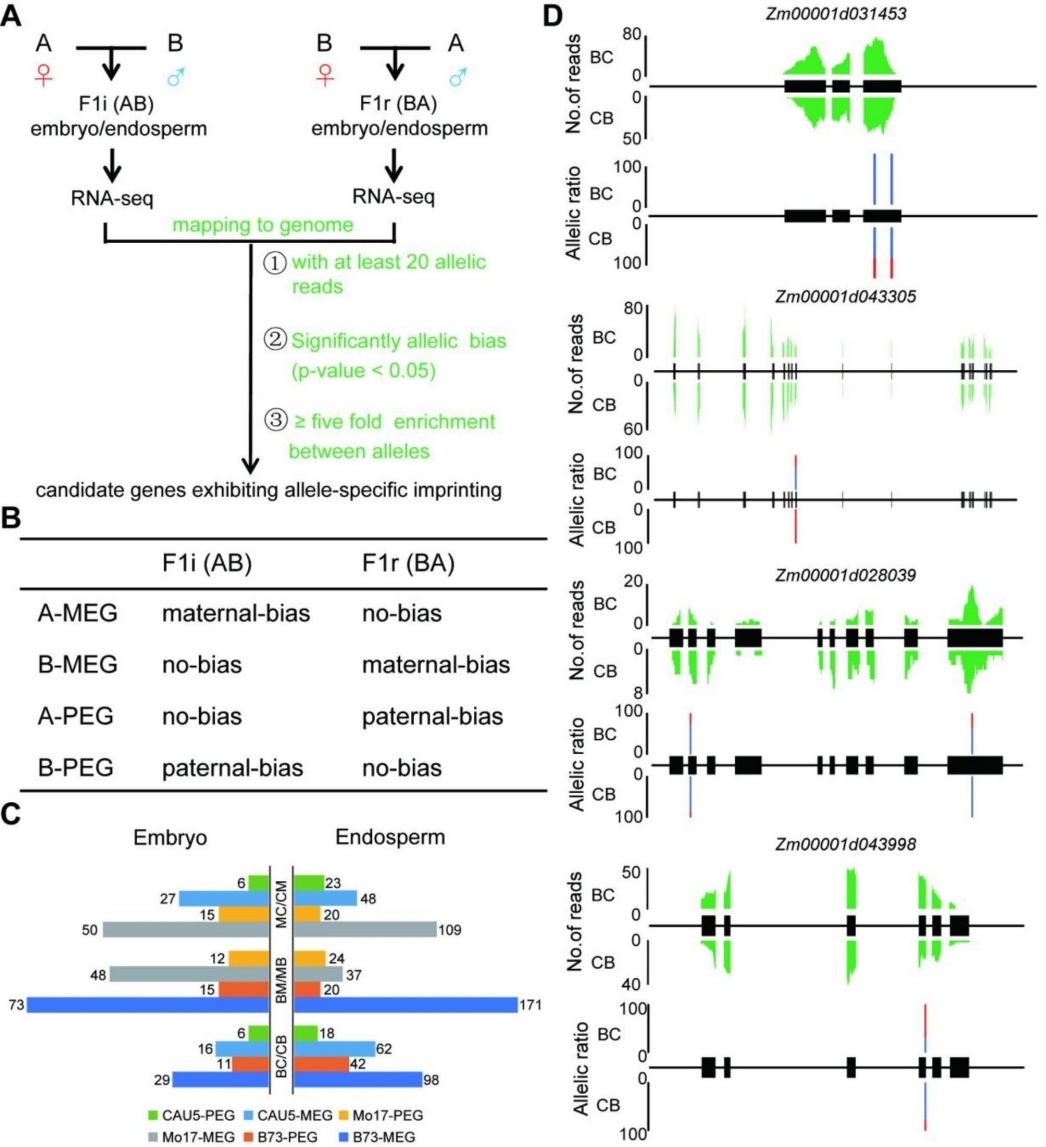
Identification and examples of ASIGs. (**A**) The pipeline to identify genes exhibiting allele-specific imprinting. A and B represent two inbred lines in maize. F1i (AB) represent that F1 hybrid from the inbred A (maternal line) crossing with inbred B (paternal line). F1r (BA) represent that F1 hybrid from the inbred B (maternal line) crossing with inbred A (paternal line). (**B**) The denoted abbreviation for ASIG showing maternal/paternal bias. A-MEG is maternally biased expression in A x B (AB), but is a no-biased expression in B x A (BA). B-MEG is a maternally biased expression in B x A (BA), but is no-biased expression in A x B (AB). A-PEG is paternally biased expression in B x A (BA), but is no-biased expression in A x B (AB). B-PEG is maternally biased expression in A x B (AB), but is no-biased expression in B x A (BA). (**C**) The number of ASIGs in the embryo and endosperm of three reciprocal hybrids (BC/CB, BM/MB and MC/CM). (**D**) The expression profile of four ASIGs identified in BC/CB endosperm. Gene expression levels are shown in green. The percentages of allelic reads for specific SNP sites are shown, with red lines for the paternal allele and blue lines for the maternal allele

In this study, a combination of statistical tests and filters of parental bias was used to classify candidate genes exhibiting allele-specific imprinting in maize embryo and endosperm (Fig. 1A and Methods). As illustrated in Fig. 1B, genes that exhibit allele-specific imprinting will only be maternally/paternally biased when a particular inbred line is the male or female parent. Under the criteria, a total of 62, 98 and 148 genes exhibited allele-specific imprinting in embryos from BC/CB, MC/CM, and BM/MB crosses, respectively (Fig. 1C; Additional file 1–3: Table S1-S3). A total of 220, 200 and 252 genes exhibited allele-specific imprinting in endosperm from BC/CB, MC/CM and BM/MB cross, respectively (Fig. 1C; Additional file 1–3: Table S1-S3). These ASIGs were further classified according to parental bias (Fig. 1C). For example, in the BC/CB endosperm, 62 C-MEGs could be an MEG in the CAU5 × B73 (CB) cross but are biallelically expressed in the B73 × CAU5 (BC) cross. Here, the expression profile of four ASIGs identified in BC/CB endosperm is illustrated in Fig. 1D-G. *Zm00001d031453, Zm00001d043305*, *Zm00001d028039* and *Zm00001d043998* are B-MEG, B-PEG, C-MEG and C-PEG in BC/CB endosperm, respectively. For example, in *Zm00001d031453* (B73-MEG), all SNPs exhibited significantly material bias in BC endosperm, compared to SNPs in CB endosperm showing a normal 2:1 ratio of maternal allele to paternal allele.

Using Circos to show genomic locations, ASIGs identified in three hybrid crosses were evenly distributed on all chromosomes without obvious location preference (Additional file 8: Fig. S1). The average distance between ASIGs was 7.43 Mb in BC/CB, 7.19 Mb in MC/CM, and 5.35 Mb in BM/MB, respectively. Most of the candidate ASIGs are not organized in physically colocalized clusters in the maize genome. There are five, one, and four pairs of candidate ASIGs located close to each other that we term clusters (Additional file 4: Table S4).

### Comparison of ASIGs between tissues and hybrids

**Fig. 2:**
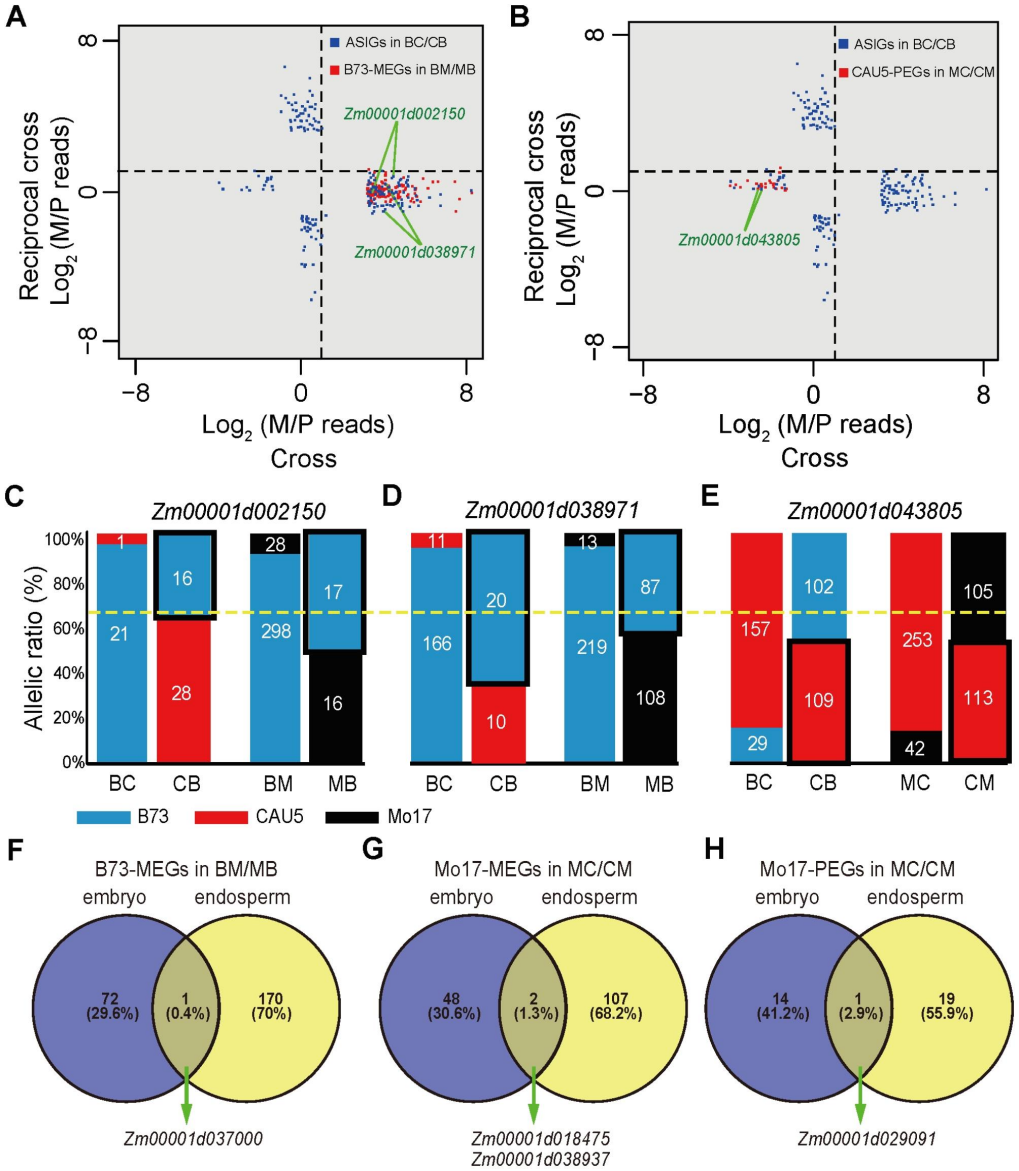
Comparison of ASIGs between tissues or hybrid crosses. (**A**) Proportion of maternal (M) and paternal (P) reads for ASIGs identified in the endosperm of BC/CB and BM/MB. The blue and red dots represented that all ASIGs in BC/CB and B-MEGs in BM/MB, respectively. (**B**) Proportion of maternal (M) and paternal (P) reads for ASIGs identified in the endosperm of BC/CB and MC/CM. The blue and red dot represented that all ASIGs in BC/CB and C-PEGs in MC/CM, respectively. (**C-E**) Expression patterns for three ASIGs. *Zm00001d002150* is a B-MEG in endosperm from BC/CB and BM/MB (**C**). *Zm00001d038971* is a B-MEG in endosperm from BC/CB and BM/MB (**D**). *Zm00001d043805* is a C-PEG in endosperm from BC/CB and MC/CM (**E**). For each bar, the upper portion represents the proportion of paternal expression, and the lower portion represents the proportion of maternal expression. The blue, red, and black bar represent the B73, CAU5 and Mo17 alleles, respectively. The yellow dashed line across the plot represents the expected biallelic ratio of 66% maternal reads. Black boxes highlight the non-imprinted allele. (**F-H**) Venn diagram analysis of ASIGs identified in the embryo and endosperm of one hybrid cross

As each allele was included twice as the maternal or paternal parent in our experimental design, we were able to identify loci that consistently showed maternal or paternal bias when a particular strain was the male or female parent. As a result, we identified two genes (*Zm00001d002150* and *Zm00001d038971*) that showed maternally biased expression in the endosperm when the B73 inbred line was used as the female parent (BC and BM cross), but showed biallelic expression in the endosperm of CB and MB (Fig. 2A and C-D). One gene (*Zm00001d043805*) showed paternally biased expression in endosperm when the CAU5 inbred line was used as the male parent (BC and MC), but showed biallelic expression in the endosperm of CB and CM (Fig. 2B and E). Therefore, few ASIGs showed consistent patterns of allele imprinting in different hybrid crosses.

Then, we examined the genes that showed consistent patterns of allelic imprinting across tissues in a hybrid cross. As visualized in the Venn diagram, only four ASIGs were consistent in both the embryo and endosperm of three hybrid crosses (Fig. 2F-H). In BM/MB crosses, a gene (*Zm00001d037000*) showed maternally biased expression when the inbred line B73 was used as the female parent in embryo and endosperm (Fig. 2F). In the MC/CM crosses, two genes (*Zm00001d018475* and *Zm00001d038937*) showed maternally biased expression in the embryo and endosperm when the Mo17 inbred line was used as the female parent (Fig. 2G). In the MC/CM crosses, one gene (*Zm00001d029091*) showed paternally biased expression when the Mo17 inbred line was used as the male parent in the embryo and endosperm (Fig. 2H).

### ASIGs tend to be expressed in various tissues

We examined whether ASIGs were embryo-specific/endosperm-specific in expression or if they were also expressed in other parts of the maize plant. Based on the gene expression levels collected from different tissues from published data [43], we found that most ASIGs were expressed in various tissues of the maize plant, indicating that these genes may have functions in the development of several plant tissues (see Methods, Additional file 9: Fig. S2). Additionally, seven genes (*Zm00001d030707*, *Zm00001d047250*, *Zm00001d018254*, *Zm00001d024810*, *Zm00001d045787*, *Zm00001d024652*, *Zm00001d044385*) were specifically expressed in the endosperm. It should be noted that *Zm00001d047250* (B-PEG in endosperm of BC/CB) and *Zm00001d024810* (B-MEG in endosperm of BC/CB) were PLATZ transcription factor 14 (*platz14*) and MYB-related transcription factor 33 (*mybr33*).

### The GO enrichment of ASIGs

To explore the function of ASIGs in the kernel development of maize, we performed a GO analysis to examine the functional distribution of the ASIGs identified in our study (Additional file 10: Fig. S3). For ASIGs identified in the embryo, five GO categories exhibited significantly higher functional enrichments compared to the set of background genes (P ≤ 0.01). These groups included response to acid chemicals, response to inorganic substances, symporter activity, signaling, and protein phosphorylation. For the ASIGs identified in endosperm, eight categories of GO exhibited significantly higher functional enrichments (Fig. S3; P ≤ 0.01). These functional categories indicated the involvement of ASIGs in intercellular nutrient transport, signaling pathways, and transcriptional regulation of kernel development.

The ASIG category (maternal bias) was specifically enriched in the transmembrane transport, substrate-specific channel activity, and symporter activity groups (Additional file 10: Fig. S3). The paternal bias ASIGs were enriched in the category representing various forms of molecular interactions, such as zinc ion binding, protein binding, and protein phosphorylation (Additional file 10: Fig. S3). Two groups of ASIGs enriched in different functional pathways could play specific roles in regulating kernel development, while ASIGs (maternal bias) may be involved in nutrient transport and hormone signaling, and ASIGs (paternal bias) are likely to be involved in the regulation of gene transcription.

### Kernel phenotype analysis of an ASIG Zm00001d030305 transgenic line

**Fig. 3:**
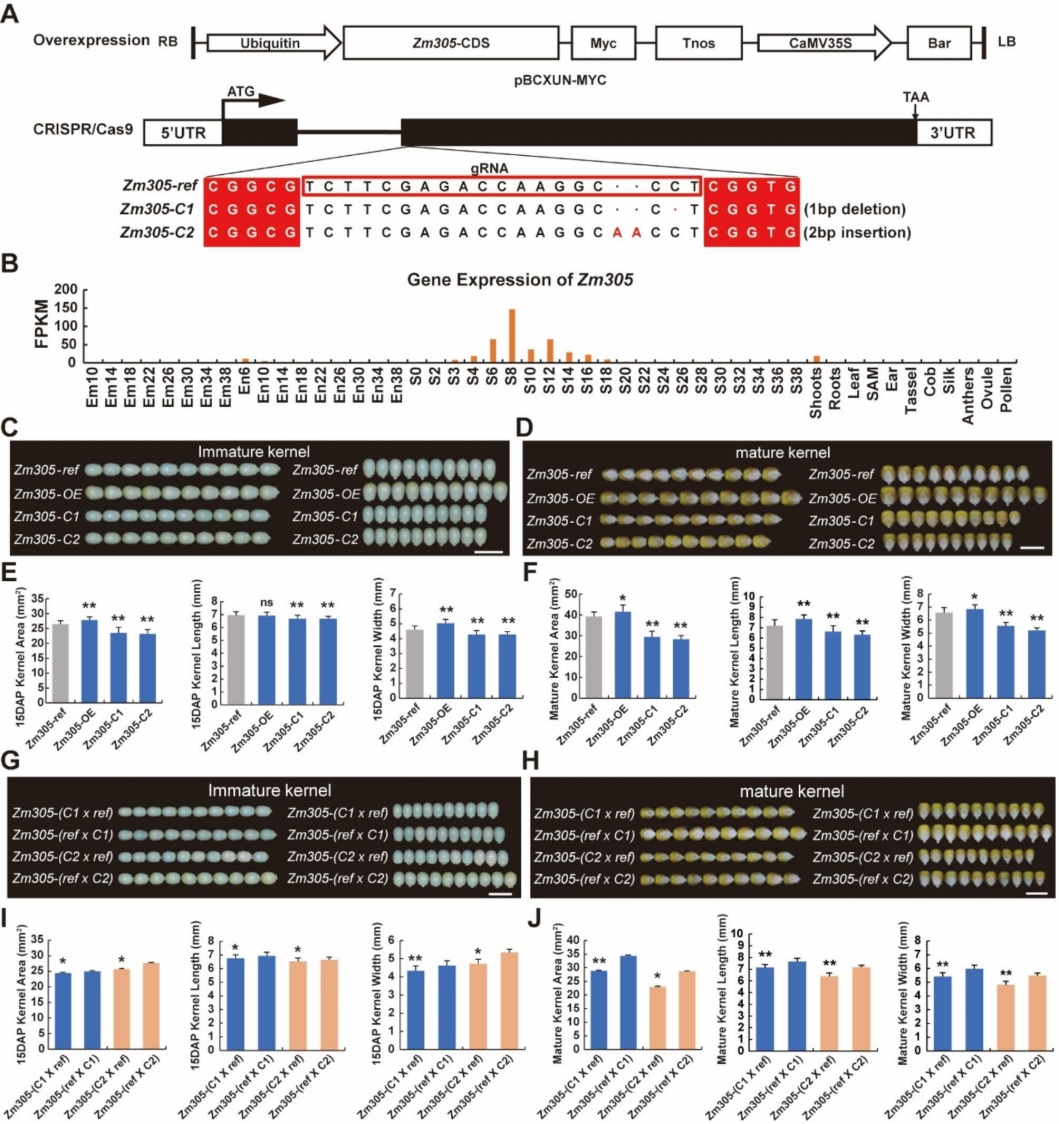
Phenotype analysis of***Zm305*** (**A**) Carrier structure of the *Zm305* overexpression and CRISPR/Cas9 lines. (**B**) Expression pattern of *Zm305*. (**C** and **D**) Immature and mature kernel phenotypes of transgenic receptor, overexpression line, and two transgenic lines. (**E** and **F**) Comparison of the immature and mature kernel area of the transgenic receptor, overexpression line, and two transgenic lines. (**G** and **H**) Immature and mature kernel phenotypes of two reciprocal crosses that were used as a transgenic receptor hybrid with two transgenic lines. (**I** and **J**) Comparison of the immature and mature kernel area of two reciprocal crosses that were used as a transgenic receptor hybrid with two transgenic lines. The primers used in the mutant identification process are listed in Table S5. The error bars indicate ± SD. Significant differences were analyzed by two-tailed Student’s t tests (ns, not significant; *P < 0.05; **P < 0.01). The gRNA was short for guild RNA; DAP was short for day after pollination; FPKM was short for fragments per kilobase per million

To further analyze the function of ASIGs in the maize kernel development, we selected an ASIG, *Zm00001d030305* (*Zm305*), which is maternally specific expression in the endosperm of Mo17 × CAU5 (MC), but is biallelically expressed in endosperm of CAU5 × Mo17 (CM) (Table S3), for further phenotype analysis by gene editing and overexpression. First, we used transgene technology to create an overexpression line, denoted as Zm305-OE (Additional file 11: Fig.S4 A-B). Then, two homozygous knockout lines from *Zm305* with a 1 bp deletion and a 2 bp insertion in the target sites were identified by PCR amplification and sequencing analysis (Fig. 3A, Table S5).

Considering that *Zm305* was highly expressed in kernels (Fig. 3B), we focused on the kernel phenotypes of the two knockout lines, overexpression lines, and the transgenic receptor line during kernel development (Fig. 3C-F). Both the length and width of immature and mature kernels in the two knockout lines showed a significant decrease compared to those of the transgenic receptor line (p value < 0.01, Student’s test) (Fig. 3C-D). In contrast, in the *Zm305* overexpression lines, the immature and mature kernel areas were significantly larger than those of the control lines, which further demonstrates that *Zm305* function may be involved in the maize kernel development. Further comparison of the kernel width and length between the knockout lines and the transgenic receptor line showed that the kernel length and width of the two knockout lines were significantly decreased compared with those of the transgenic receptor line, while they were both significantly increased compared with those of the transgenic receptor line in the overexpression lines, which showed that the length and width of the kernel could both affect the kernel area (Fig. 3E-F).

To explore whether the kernel phenotype of two knockout lines crossed with the transgenic receptor line conforms to the imprinting patterns, we compared the immature and mature kernel phenotypes of two reciprocal crosses, in which two knockout lines and the transgenic receptor line were used as parents, respectively. As shown in Fig. 3G-H, the kernel size of the crosses showed a significant decrease compared with the corresponding reciprocal cross when two knockout lines were used as a maternal to cross with the transgenic receptor line, and this trend of change was consistent with the phenotypic imprinting pattern of maternal imprinted genes. Interestingly, the kernel length and width data for two reciprocal crosses also verified that the significant difference in kernel size between reciprocal crosses might be affected by both kernel length and width (Fig. 3I-J).

## Discussion

### Conservation of ASIGs within maize

By transcriptome analysis, a total of 305 ASIGs were identified in the embryo and 655 ASIGs in the endosperm of three maize F1 hybrids. About half of ASIGs identified in one crosses can be allelically analyzed in other crosses. In this study, only three ASIGs (*Zm00001d002150*, *Zm00001d038971*, and *Zm00001d043805*) were consistently showed maternal or paternal bias when a particular strain was male or female parent.

In previous work, the 17 genes (8 PEGs and 9 MEGs) that showed imprinting variation were identified in the maize five reciprocal hybrids including B73 × Mo17 and Mo17 × B73 (denoted as BM/MB), B73 × Ki11 and Ki11 × B73 (denoted as BK/KB), Mo17 × Ki11 and Ki11 × Mo17 (denoted as MK/KM), B73 × Oh43 and Oh43 × B73 (denoted as BO/OB), Mo17 × Oh43 and Oh43 × Mo17 (denoted as MO/OM) [19]. Then, we investigated the allelic expression of the 17 genes in our data. In endosperm, we found that two genes (*Zm00001d038075* and *Zm00001d043878*) were shown to be conserved imprinting variation in our data. As reported in the previous work, *Zm00001d038075* was a B-MEG in the BM/MB endosperm and an M-MEG in the MO/OM endosperm [19]. In our data, *Zm00001d038075* was a B-MEG in BM/MB endosperm, and we did not identify *Zm00001d038075* as an M-MEG in the MC/CM hybrid due to the lack of SNPs between Mo17 and CAU5. Furthermore, in previous work, *Zm00001d043878* was an M-PEG in the MK/KM [19]. In our data, *Zm00001d043878* was an M-PEG in the MC/CM endosperm. Therefore, our results indicated that the degree of conservation of ASIGs was lacking within maize. Of course, some imprinted genes were stage-specifically expressed or stage-specifically imprinted during endosperm development. Meanwhile, the different developmental program of the endosperms from reciprocal hybrids possibly exist. Hence, generating RNA-seq data from multiple time points of maize endosperm enable us to accessibly investigate the allele-specific imprinting.

### The regulatory mechanism and potential function of ASIGs

Imprinted genes are frequently involved in energy metabolism and seed development. The theory of parent-offspring conflict has more supporting evidence than the other hypotheses to explain the evolution of imprinted genes [23, 44], suggesting that the function of imprinted genes should be enriched in the synthesis and transport of nutrients. However, analysis of the function of allele-specific imprinted genes is limited. Allele-specific imprinting represented genes that are imprinted specifically in one strain and not the other due to differences in control over endosperm growth and development. As known, there are three theories for the evolution of genomic imprinting: sexual antagonism theory, maternal-offspring coadaptation theory and parental conflict theory [45]. Under the parental conflict theory, inbreds producing small seeds could be due to more maternalized while inbreds producing larger seeds could be due to more paternalized. One allele-specific imprinted gene, HDG3 (the class IV homeodomain-leucine zipper (HD-ZIP) transcription factor), is a PEG in Cvi x Col crosses but is biallelically expressed in Col x Cvi [46]. The gain in HDG3 imprinting was associated with earlier endosperm cellularization and changes in seed weight. Therefore, the function of ASIGs identified in our study will be further studied in the future. Three ASIGs (Zm00001d002150, Zm00001d038971, and Zm00001d043805) consistently showing maternal or paternal bias are being prioritized to investigate their function. Zm00001d043805 was annotated as an E3 ubiquitin ligase. The homolog of Zm00001d038971 in Arabidopsis is AT1G28380, which is a MAC/Perforin domain-containing protein involved in the negative regulation of salicylic acid-related defense responses and cell death programs. In addition, ASIGs specifically expressed in embryo or endosperm are also recommended for preferential function analysis. For example, Zm00001d047250 (platz14) is a PLATZ-transcription factor [47]. And FL3 (ZmPLATZ12) is specifically expressed in starchy cells of maize endosperm and functions as a modulator of the RNAPIII transcription complex consistent with the highly abundant synthesis of tRNA and 5 S rRNA in the maize endosperm [35].

To further detect the potential roles of ASIGs belonging to the GO term “nucleotide binding”, we investigated the interaction of these ASIGs using the STRING database. The results showed that the two interaction networks were identified and the network exhibited complex functional relationships (Additional file 12: Fig. S5). For instance, Zm00001d032867 has the most interactions with other nine genes. Zm00001d032867 is annotated as a phosphoglycerate kinase, which is a cytosolic enzyme. The Zm00001d003659 has the most interactions with other four genes in the other network. The Zm00001d003659 is annotated as a serine/threonine-protein kinase. As reported, the serine/threonine protein kinase encoding gene KERNEL NUMBER PER ROW6 (KNR6) can significantly influence grain yield [48]. These results suggest that ASIGs are potentially involved in several biological processes of seed development (Additional file 12: Fig. S5). Further exploring the function of these ASIGs for seed development in more genetic backgrounds will be required to determine if differences in imprinting contribute to seed phenotypes among backgrounds.

Natural variation in DNA methylation is associated with imprinting variation. The mechanism and function of HDG3 allele-specific imprinting indicated that epigenetic variation alone is sufficient to explain the imprinting variation and demonstrate that epialleles can underlie the variation in the phenotypes of seed development. Variation in DNA methylation was also observed between BM/MB and MC/CM endosperm. Further study of the relationship between variation in DNA methylation and expression of ASIGs should be performed in future work.

### Internal factors influenced the defective kernel phenotype

In our study, knocking out *Zm305* resulted in a smaller kernel size. *Zm305* was annotated as a proline-rich protein, which is involved in cell-wall signaling, plant development, and stress responses. In rice, a glycine- and proline-rich protein (*OsGPRP3*) regulates the grain size and shape by influencing the accumulation of storage protein and lipids [49]. To explore the internal factors of *Zm305* that affect the kernel size, we further detected protein content, oil content, starch content, soluble sugar content, amino acid content, amylose and amylopectin content, and 17 amino acid content between *Zm305* mutants and wild-type kernels. Only the soluble sugar content, the amylose and amylopectin content, and the 17 amino acid content showed a difference between mutant and wild-type kernels. In the previous study, the soluble sugar content was shown to affect grain length and grain thickness, and there was a positive correlation with grain width in the mature seed period [50]. A high soluble sugar content in the mature period causes an obvious decrease in amylose content, which could lead to a phenotype with defective kernel development [50]. As shown in Fig. S6A, the soluble sugar content of the two *Zm305* mutant lines was all higher than that of the control lines, which was consistent with previous studies. Furthermore, the amylose content of the two *Zm305* mutant lines increased significantly, although the total starch content was not significant compared to the control lines (Additional file 13: Fig. S6B-C). Few of the 17 amino acids contents were significantly different between the mutants and control lines, such as Alanine, Histidine, L-isoleucine, Leucine, Phenylalanine, Serine, and Valine (Table S6). Therefore, we speculated that ASIGs may influence the kernel development process by regulating the soluble sugar content of maize kernels. However, since the amylose content of the kernel also showed a remarkable difference in our results, we do not rule out the possibility that the mutant kernel phenotype is influenced by other factors, and the detailed molecular regulatory mechanism still needs further research.

## Conclusions

In this study, three maize inbred lines (B73, Mo17 and CAU5) were chosen to generate three reciprocal crosses, BC/CB, MC/CM, and BM/MB. By transcriptome analysis, we identified several hundred ASIGs in the maize kernel. The conservation of these ASIGs was relative lacking within maize. GO analysis indicated that ASIGs were involved in intercellular nutrient transport, signaling pathways, and transcriptional regulation of kernel development. Further research on the function of ASIGs will contribute to our understanding of the relationship between intraspecific variation in imprinting and seed development variation.

## Materials and methods

### Plant materials collection

Six hybrid crosses were obtained from the inbred lines B73, Mo17 and CAU5 in the summer of 2021 at the experimental station of Shenyang Agriculture University in Shenyang, Liaoning, B73(♀) × Mo17(♂), Mo17(♀) × B73(♂), B73(♀) × CAU5(♂), CAU5(♀) × B73(♂), CAU5(♀) × Mo17(♂), Mo17(♀) × CAU5(♂). The ears and tassels of the B73, Mo17 and CAU5 lines were bagged with kraft paper one day prior to pollination. The next day, each paper bag was patted to collect pollen from one parent, which was used to pollinate the ear of the other parent. The maize endosperm at 11 days after pollination have finished the switches from mitosis to endoreduplication and starts to be filled with starch and storage proteins. The corresponding embryo at 11 days after pollination were collected. At 11 days after the pollination, the embryos and endosperm of six reciprocal crosses (BM, MB, BC, CB, MC, and CM) were collected. The embryos used for RNA-seq analysis are diploid which firstly identified by KASP markers, and the individuals of haploid embryos are removed. The KASP markers used in this study has been listed in supplemental table S7. In this study, BM /MB represents the crosses of B73 × Mo17 and Mo17 × B73, BC /CB represents the crosses of B73 × CAU5 and CAU5 × B73, and MC /CM represents the crosses of Mo17 × CAU5 and CAU5 × Mo17.

### Library construction for RNA-seq

The protocal for RNA-seq library construction of the embryo and endosperm samples were the same as in our previous work [42]. Total RNA was extracted using a Quick RNA Isolation Kit (Huayueyang Biotechnology of Beijing). RNA-seq libraries were constructed and sequenced using the Illumina NovaSeq 6000 platform. 150 bp paired-end reads were generated for each library. Three biological replicates were set up for each of the embryos and endosperm from six reciprocal crosses (BM, MB, BC, CB, MC, and CM). An average of 28 million pair-end reads for each replicate was used for the further analysis.

### Identification of ASIGs

The method for clean reads mapping and reads being separated into maternal or paternal allele was the same as in our previous work [42]. A total of 1,669,940 SNPs in the BC/CB cross, 1,588,429 SNPs in the MC/CM cross, and 1,299,229 SNPs in the BM/MB cross to distinguish parental alleles. Maternal and paternal read counts of each gene were summed. First, the ratio of maternal/paternal reads was calculated for all genes with ≥ 20 informative reads at SNP sites in embryo and endosperm from BC/CB, BM/MB and MC/CM. As result, a total of 13,019, 12,768 and 15,628 were allelically analyzed genes in the embryo from BC/CB, MC/CM and BM/MB. And 11,620, 10,970 and 14,711 were allelically analyzed genes in the embryo from BC/CB, MC/CM and BM/MB. Then, maternal/paternal reads of genes were detected with the deviation of the maternal: paternal ratio from the theoretical ratio using χ2 test (1:1 in the embryo and the 2:1 in the endosperm). Finally, read counts from one parental allele were used to identify ASIGs at least five-fold higher than read counts from another parental allele. Candidate ASIGs will only be maternal/paternal bias when a particular inbred is the male or female parent. These ASIGs were further classified depending on parental bias. For example, in embryos from BC/CB, B73-MEGs were identified with significantly allelic bias (χ2 < 0.05) and > 83.3% of the transcripts derived from the maternal allele in B73 × CAU5, but without significantly allelic bias (χ2 > 0.05) in CAU5 × B73; CAU5-MEGs were identified with significantly allelic bias (χ2 < 0.05) and > 83.3% of the transcripts derived from the maternal allele in CAU5 × B73, but without significantly allelic bias (χ2 > 0.05) in B73 × CAU5; B73-PEGs were identified with significantly allelic bias (χ2 < 0.05) and > 83.3% of transcripts derived from the paternal allele in CAU5 × B73, but without significantly allelic bias (χ2 > 0.05) in B73 × CAU5. The CAU5-PEGs were identified with significantly allelic bias (χ2 < 0.05) and > 83.3% of the transcripts derived from the paternal allele in B73 × CAU5, but without significant allelic bias (χ2 > 0.05) in CAU5 × B73. In endosperm, imprinted expression was also identified with a significant allelic bias (χ2 < 0.05), with > 90.9% of the transcripts from the maternal allele or > 71.4% of the transcripts from the paternal allele.

### GO term enrichment and functional category analysis

GO analysis of ASIG was performed by AgriGO v2.0 (accessed on 2 June 2022) [51]. GO terms were retained with significant (p-value < 0.01) enrichment compared to the set of background genes.

### Genetic transformation of maize

We prepared overexpression constructs for the genetic transformation of *Zm00001d030305* (*Zm305*). Full-length CDS (without stop codon) of *Zm305* was amplified from *Zm305* cDNA and cloned into the binary vector pBCXUN-MYC to generate the pOE *Zm305*-MYC construct driven by the ubiquitin promoter. Transformations using the overexpression construct were introduced into the maize receptor line KN5585 via Agrobacterium-mediated transformation [52]. For the CRISPR/Cas9 gene-editing construct, one 19-bp sequence from the second exon of *Zm305* was selected as a guide RNA (gRNA) and introduced into the pBUE411 vector as previously described [53]. For transformations using the CRISPR/Cas9 construct, two homozygous knockout lines from this gene with insertions or deletions in the target sites were identified from independent positive transgenic lines (T0) via PCR amplification and sequencing analysis (Table S5). Independent positive transgenic lines were obtained and self-pollinated to generate homozygous progenies for kernel phenotype analysis.

### The method of measuring kernel area

One third of the kernels in the middle of the crossed ears of Zm305-C1 x WT, WT x Zm305-C1, Zm305-C2 x WT, WT x Zm305-C2, Zm305 selfing ear, and WT selfing ear at 15 DAP and 45 DAP were separated and imaged under a light microscope (Olympus, Tokyo, Japan) one by one. Image J software was used to measure the area of each kernel. At least ten ears were used as biological replicates for phenotype analysis in each cross.

### Components contents measurement

The total amino acid and 17 amino acid content were measured using the INFRATEC Nova 17,001,854 S Near infrared grain analyzer. Nearly 100 kernels of each line were randomly selected to analyze the above character for one biological repeat, each line needs three biological repeats.

The starch and amylose content were determined using the method described previously [54].

The soluble sugar content was analyzed using the steps as described previously [55].

### Primers

All primers used in this study are listed in Table S5.

### Electronic supplementary material

Below is the link to the electronic supplementary material.

Supplementary Material 1

Supplementary Material 2

## Data Availability

Sequence data from this study can be found in the Sequence Read Archive at NCBI (SRA; http://www.ncbi.nlm.nih.gov/sra) under accession numbers and PRJNA765150.

## Abbreviations

BM and MB: Crosses of B73 × Mo17 and Mo17 × B73, respectively
BC and CB: Crosses of B73 × CAU5 and CAU5 × B73, respectively
MC and CM: Crosses of Mo17 × CAU5 and CAU5 × Mo17, respectively
ASIGs: Genes exhibiting allele-specific imprinting
GO: Gene ontology
DAP: Days after pollination
WT: Wild type
