# Supplementary material for: Transcriptome-wide identification and characterization of genes exhibit allele-specific imprinting in maize embryo and endosperm
Source: BMC Plant Biol. 2023 Oct 6;23:470. doi: 10.1186/s12870-023-04473-8 (PMC10557216; doi:10.1186/s12870-023-04473-8)
Supplement: Supplementary file 1 — Supplementary Material 1 [file 12870_2023_4473_MOESM1_ESM.docx]

# Supplementary Information

**Additional file 1: Table S1.** The ASIGs identified in the BC/CB embryo and endosperm.

**Additional file 2: Table S2.** List of ASIGs identified in the BM/MB embryo and endosperm.

**Additional file 3: Table S3.** List of ASIGs identified in the MC/CM embryo and endosperm.

**Additional file 4: Table S4.** Clusters of ASIGs in this experiment.

**Additional file 5: Table S5.** Primers used in this experiment.

**Additional file 6: Table S6.** Comparison of 17 amino acids content between the *Zm305* mutant and transgenic receptor line.

**Additional file 7: Table S7.** KASP markers used for ploid detection in the experiment

**Additional file 8: Figure S1.** Chromosomal distribution of ASIGs identified in three reciprocal crosses.

**Additional file 9:** **Figure S2.** The expression of the ASIGs in maize tissues.

**Additional file 10:** **Figure S3.** The GO-enriched term of ASIGs in embryo and endosperm.

**Additional file 11: Figure S4.** The PCR identification of bar gene and gene expressed level of Zm305 in overexpression lines and control lines.

**Additional file 12: Figure S5.** The interaction networks of ASIGs belonging to the GO term “nucleotide binding”.

**Additional file 13:** **Figure S6.** Comparison of starch content between the transgenic receptor and two transgenic lines.

**
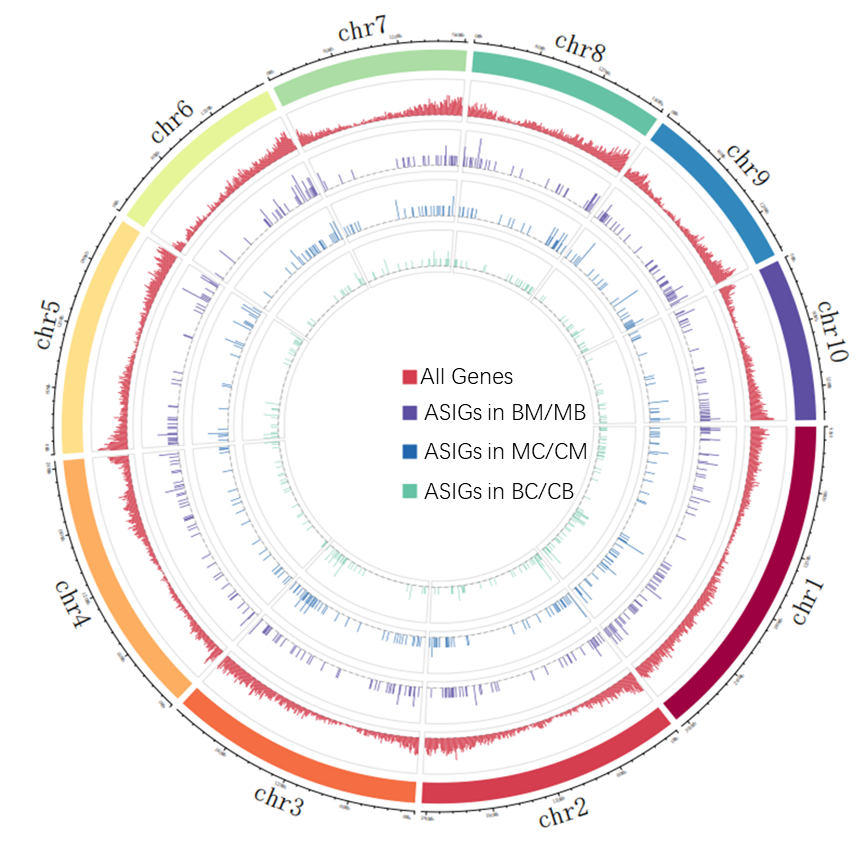
**

**Fig. S1:** Chromosomal distribution of ASIGs identified in three reciprocal crosses.


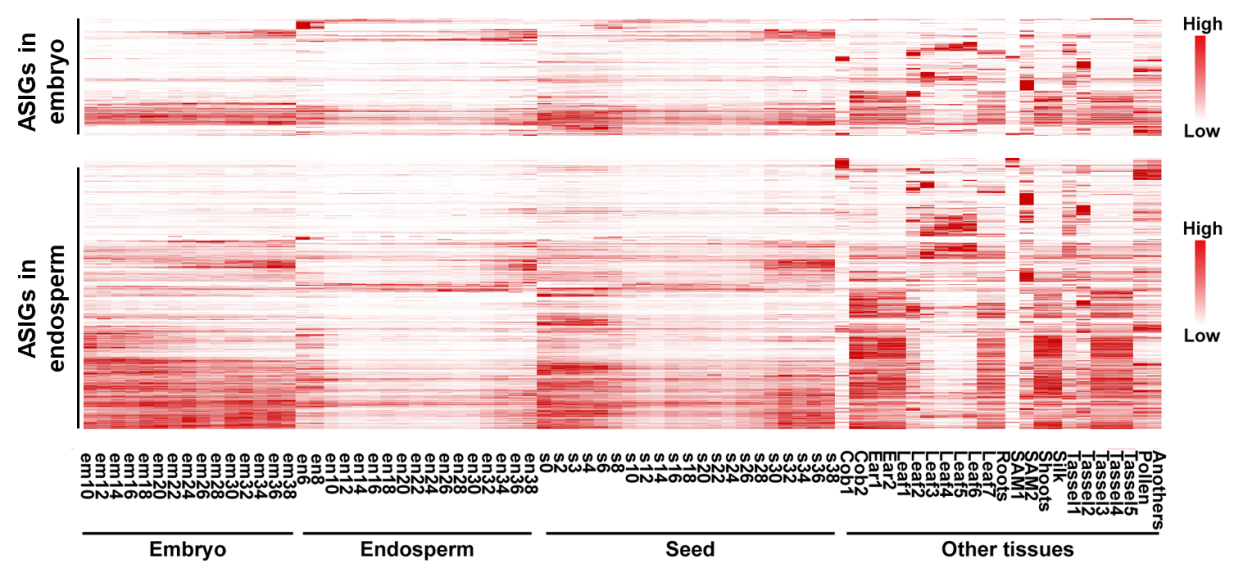


**A**

**B**

**Fig. S2:** The expression of the ASIGs in maize tissues. (A) The expression of the ASIGs identified in the embryo of BM/MB, BC/CB, and MC/CM. (B) The expression of the ASIGs identified in the endosperm of BM/MB, BC/CB, and MC/CM.


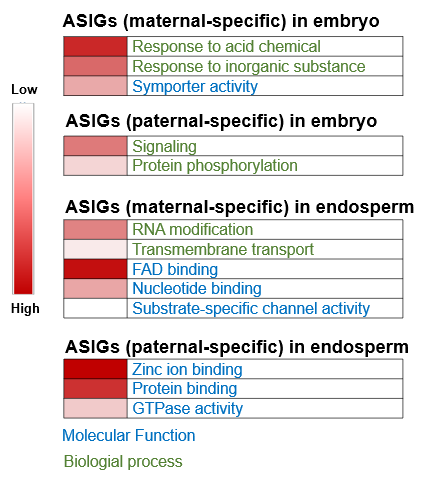


**Fig. S3:** The GO-enriched term of ASIGs in embryo and endosperm.


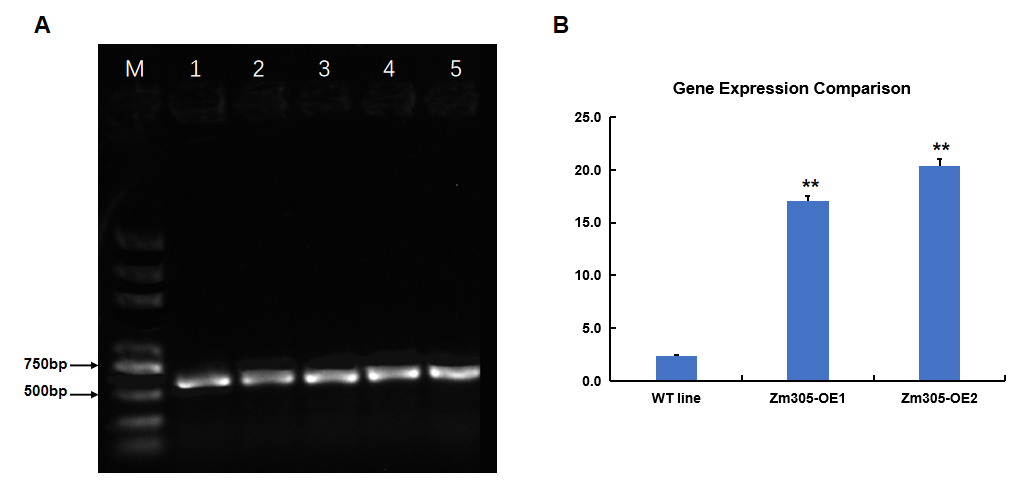


**Fig. S4:** PCR identification of bar gene and gene expressed level of Zm305 in overexpression lines and control lines. (A) PCR identification of bar gene in overexpression lines; (B) gene expressed level of Zm305 in overexpression lines and control lines.


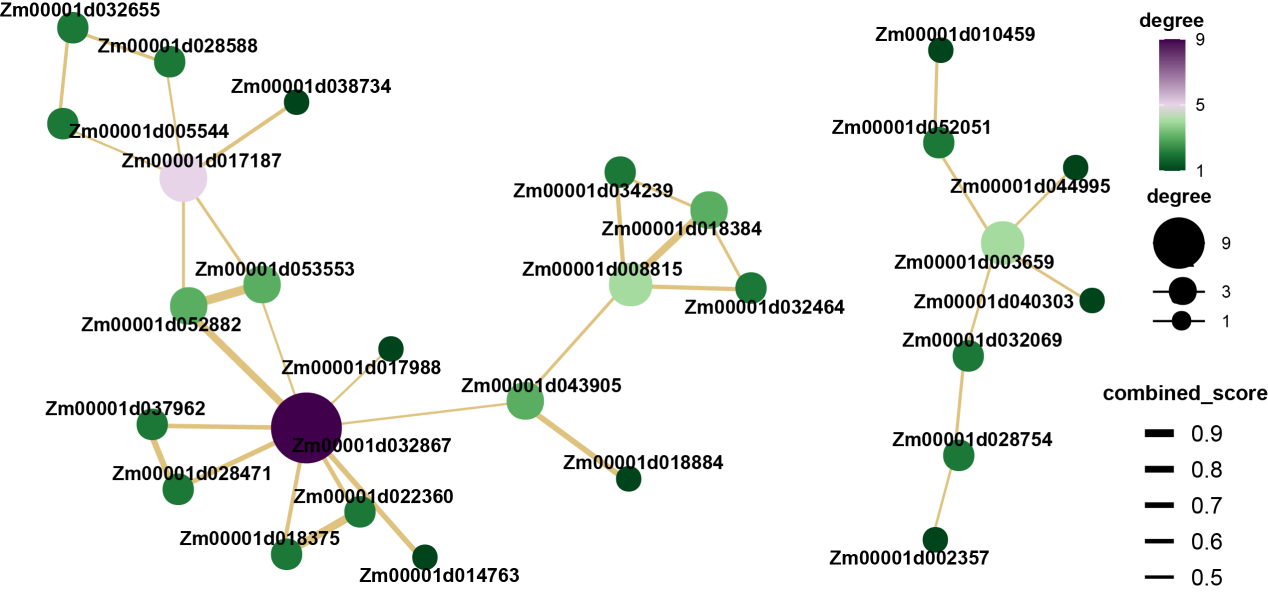


**Fig. S5:** The interaction networks of ASIGs belonging to the GO term “nucleotide binding”.

**
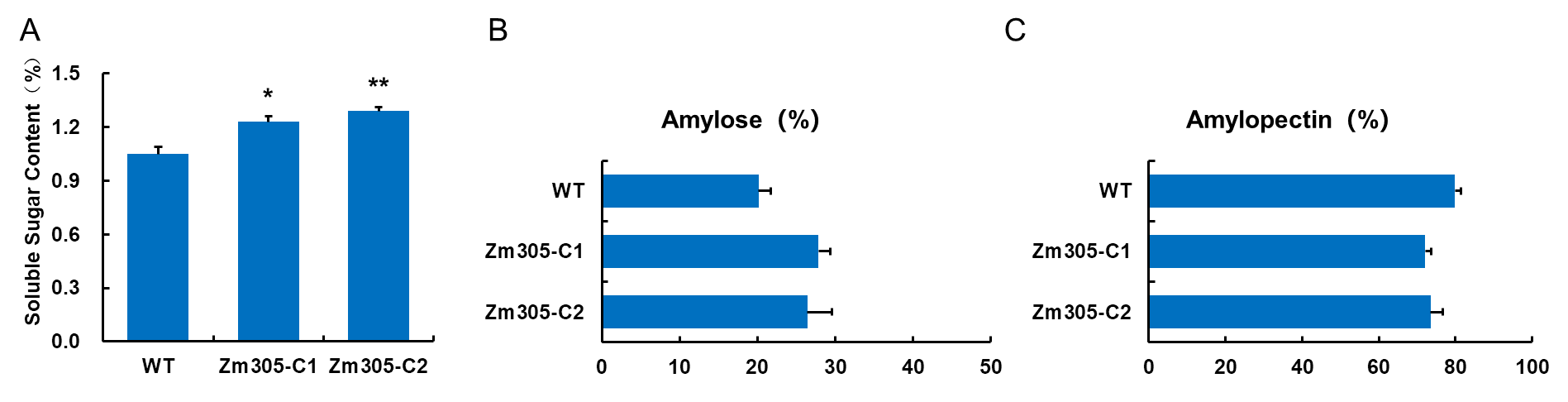
**

**Fig. S6:** Comparison of starch content between transgenic receptor and two transgenic lines.

(A) Comparison of soluble sugar content between transgenic receptor and two transgenic lines. (B) Comparison of amylose content between transgenic receptor and two transgenic lines. (C) Comparison of amylopectin sugar content between transgenic receptor and two transgenic lines.
